# Depletion of the Aryl Hydrocarbon Receptor in MDA-MB-231 Human Breast Cancer Cells Altered the Expression of Genes in Key Regulatory Pathways of Cancer

**DOI:** 10.1371/journal.pone.0100103

**Published:** 2014-06-16

**Authors:** Gennifer Goode, Siddharth Pratap, Sakina E. Eltom

**Affiliations:** 1 Department of Biochemistry and Cancer Biology, Meharry Medical College, Nashville, Tennessee, United States of America; 2 Micorarray and Bioinformatics Core, Meharry Medical College, Nashville, Tennessee, United States of America; Wayne State University, United States of America

## Abstract

The aryl hydrocarbon receptor (AhR), a transcription factor that is best known for its role in mediating the toxic responses elicited by poly aromatic hydrocarbons as well as many other environmental factors; is also involved in breast cancer progression. We previously reported that stable knockdown of AhR decreased the tumorigenic properties of the highly metastatic MDA-MB-231 breast cancer cell line; whereas ectopic overexpression of AhR was sufficient to transform immortalized human mammary epithelial cells to exhibit malignant phenotypes. In the present study we investigated the genes that are differentially regulated by AhR and are controlling cellular processes linked to breast cancer. We used Affymetrix Human GeneChip 1.0-ST whole transcriptome arrays to analyze alterations of gene expression resulting from stable AhR knockdown in the MDA-MB-231 breast cancer cell line. The expression of 144 genes was significantly altered with a ≥2.0-fold change and a multiple test corrected p-value ≤0.05, as a result of AhR knockdown. We demonstrate that AhR knockdown alters the expression of several genes known to be linked to cancer. These genes include those involved in tryptophan metabolism *(KYNU)*, cell growth (*MUC1* and *IL8),* cell survival (*BIRC3* and *BCL3*), cell migration and invasion (*S100A4* and *ABI3),* multi-drug resistance (*ABCC3*) and angiogenesis (*VEGFA* and *CCL2*). The identification of the genes and pathways affected by AhR depletion provides new insight into possible molecular events that could explain the reported phenotypic changes. In conclusion AhR knockdown alters the expression of genes known to enhance or inhibit cancer progression; tipping the balance towards a state that counteracts tumor progression.

## Introduction

The aryl hydrocarbon receptor (AhR) is a ubiquitously expressed ligand-activated transcription factor that belongs to the basic-helix-loop-helix–Per-ARNT-Sim superfamily of transcription factors [1]. The AhR-mediated regulation of metabolism of polycyclic aromatic hydrocarbons (PAH) has been implicated in a variety of cancers [2], [3]. PAH-induced AhR activation increases the transcription of a variety of genes encoding phase I drug metabolizing enzymes, such as CYP1A1 and CYP1B1, and phase II drug metabolizing enzymes, such as glutathione-S-transferase A1 (GSTA1) and UDP glucuronosyl transferase 1A2 (UGT1A2). In particular the CYP enzymes promote metabolic activation of PAHs increasing the levels of PAH-DNA adducts which are associated with cancer initiation. Additionally, PAH-induced AhR activation was shown to play a role in cancer promotion and progression [4], [5].

Although expressed in normal tissues, elevated AhR expression has been reported in several cancer types including lung, breast, liver, stomach and pancreas [6], [7]. This elevated expression is associated with constitutive activation in the absence of exogenous ligand, evident by the predominant nuclear localization of AhR and induced expression of AhR responsive gene, *CYP1A1* [8], [9]. These findings suggest a role for AhR in cancer independent of exogenous ligand. In support of this notion, studies designed to mimic a constitutively active AhR showed a role for AhR in tumor promotion and progression [10], [11].

In breast cancer, elevated and constitutively active levels of AhR were found in advanced human breast tumors and breast cancer cell lines, with a strong correlation between expression of AhR and the degree of the tumor malignancy [12]. Interestingly, ectopic overexpression of AhR in immortalized normal mammary epithelial cells induced a malignant phenotype with increased growth and acquired invasive capabilities proportional to the level of AhR expressed [13]. Subsequently, we showed that knockdown of the inherently elevated levels of AhR in the highly metastatic MDA-MB-231 breast cancer cell line, decreased their tumorigenic properties both *in vitro* and *in vivo* [9]. Based on these phenotypic changes, we hypothesize that the elevated levels of AhR in advanced breast cancer are driving signaling pathways involved in cell survival, adhesion and invasiveness. In the present study we sought to identify alterations of global gene expression in MDA-MB-231 cells following stable AhR knockdown in order to determine which of the genes and signaling pathways involved in breast cancer progression are affected by the elevated AhR levels.

## Materials and Methods

### Cell Culture

The triple negative human breast carcinoma (HBC) cell line MDA-MB-231 was purchased from ATCC (Manassas, VA). Cells were cultured in the recommended medium [L-15 medium supplemented with 10% fetal bovine serum (FBS), 100 U/ml penicillin and 100 µg/ml streptomycin] in a humidified atmosphere at 37°C with 5% CO_2_. Stable knockdowns were cultured in media supplemented with 2.5 µg/ml puromycin (Sigma-Aldrich, St. Louis, MO).

### Stable Knockdown of AhR using RNA Interference

The stable knockdown of AhR gene expression in MBA-MD-231 human breast carcinoma cell line and the generation of AhR knockdown clone 8 and its Scrambled (Scr) control cells was previously described [9]. Briefly, the MBA-MD-231 cells were infected with shRNA-AhR or shRNA-Scr control retroviral particles produced in Phoenix cells. Stable lines were selected by culturing in the presence of puromycin (2.5 µg/ml). Limited cell dilution was performed on shRNA-AhR and shRNA-Scr control cells to obtain single cell clones. Clone 8 of shRNA-AhR cells, which exhibited the highest AhR knockdown (∼80%) was chosen for subsequent analyses. Multiple clones of Scr control with equivalent AhR protein expression to the parental heterogeneous Scr control cells, were pooled, expanded and used for the subsequent studies to represent the Scr control. Both clone 8 and Scr control were used in our previous studies [9] and were analyzed here for their comparative differential gene expression.

### Isolation of RNA

Total RNA was isolated using Trizol reagent (Invitrogen Life Technologies, Carlsbad, CA) and further purified using a Qiagen RNeasy kit following the provider’s instructions (Qiagen Inc, Valencia, CA). Total RNA was treated with Qiagen RNase-Free DNase I, prior to RNA being resuspended in RNase-free water and quantified by absorbance at 260 nm using a Nanodrop ND-1000 Spectrophotometer (NanoDrop Technologies, Inc, Wilmington, DE). The quality and quantity of RNA was verified with the Bioanalyzer (Agilent Technologies, Inc, Santa Clara, CA). Only RNA samples with high quality (a RIN of 8 or greater) were used for microarray analysis.

### Transcriptome Microarray Analysis

Total RNA was submitted to Vanderbilt University Microarray Shared Resources (VMSR) (Nashville, TN) for gene expression profiling using the Affymetrix Human Gene 1.0-ST whole transcriptome array (Affymetrix, Santa Clara, CA). Synthesis and labeling of complementary DNA targets, hybridization and scanning of GeneChips were carried out by VMSR on a fee for service basis. We performed transcriptome microarray data analysis using Partek Genomics Suite version 6.6 (Partek Inc., St. Louis, MO). Relative expression levels of gene transcripts were based on the average of at three clone 8 and two Scr-control replicates. Affymetrix CEL files were normalized using the Robust Multi-array Average (RMA) algorithm [14]. Transcriptome level fold changes and the significance of those changes were calculated using one way ANOVA. Significantly changed transcripts in clone 8 cells were defined as having a ≥2.0 fold expression change from Scr-controls and a Benjamini-Hochberg (BH) false discovery rate corrected *P-value* ≤0.05.

### KEGG Pathway and Gene Ontology (GO) Enrichment Analysis

The WEB-based Gene Set Analysis Toolkit (WEBGESTALT) was used in order to conduct KEGG pathway and gene ontology (GO) enrichment analysis on the transcriptome array dataset. Briefly, gene transcripts showing significant changes in expression from the transcriptome array were mapped to their corresponding KEGG pathways and GO biological processes and a hypergeometric test was used to determine significant enrichment. To correct for multiple testing, the threshold for significance of the enrichment scores used a BH false discovery rate corrected P-value <0.05 [15].

### Biological Interaction Network Construction

To populate and build a biological interaction network of the transcriptome dataset, the Michigan Molecular Interactions (MiMI) database MiMI Cytoscape plugin (version 3.2) was used. MiMI gathers and merges data from well-known protein interaction databases including BIND, DIP, HPRD, RefSeq, SwissProt, IPI, and CCSB-HI1. The Plugin also integrates other NCIBI tools for literature information, document summarization, and pathway matching [16]. The differentially expressed genes were used as the initial population nodes then MiMI was used to query for the initial nodes and their respective nearest neighbors to one degree of biological interaction. The networks were then merged for interconnections and the global interactome was visualized in Cytoscape.

### Validation Using Quantitative Reverse Transcriptase-Polymerase Chain Reaction (qRT-PCR)

RNA (1 µg) was reverse transcribed to complementary DNA (cDNA) using random hexamer primers and Moloney murine leukemia virus reverse transcriptase in presence of RNAse inhibitor (Promega, Madison, WI). Quantitative real-time PCR was then carried out in 96-well plates in a Bio-rad CFX96 Real Time System (Bio-Rad, Hercules, CA) using QuantiFast SYBR Green (Qiagen, Valencia, CA) to monitor the PCR amplification. The real-time PCR mixtures consisted of 12.5 µL of 2X QuantiFast SYBR Green master mix, template cDNA (≤100 ng), each primer (1 µM), and ddH_2_O to give a final volume of 25 µL. The following two-step cycling program was used for all PCR reactions: 95°C for 10 min, 40 cycles of (95°C, 15 sec; and 60°C, 60 sec). The specificity of each amplification reaction was verified by a dissociation curve (melting curve) consisting of 10 s incubation at 95°C, 5 s incubation at 65°C, a ramp up to 95°C. All samples were amplified in triplicates and relative quantification of the expression level of each gene was calculated using the delta CT method in CFX manager software (Bio-Rad, Hercules, CA). Ribosomal 18s was used as the endogenous reference gene. Non-template controls were included for each primer pair. Gene-specific primers were designed using Applied Biosystems Primer Express software (Life Technologies, Grand Island, NY), (Table 1).

**Table 1:** List of primers used for validation of microarray data by quantitative reverse transcriptase-polymerase chain reaction.

Gene	Primer sequence (5′–3′)
C15ORF48	F: CCTGATGAAAAGGAAGGAACTCA
	R: CCGCCGCCACAGTCA
CALB1	F: CAGAATCCCACCTGCAGTCA
	R: TGGAGCCAGATCTCGAAAAACT
CSF1	F: TGCAGCGGCTGATTGACA
	R: GATCTTTCAACTGTTCCTGGTCTACA
HLADPA1	F: GAACTCCAGCTGCCCTACAAA
	R: TGGAGTAGTTTTCACATGAAGTGAGA
KYNU	F: TCCATTGGGATCCTAGCTGTTT
	R: AGCCGGCAGCTCAAGAGAT
MRGPRX4	F: TGGCCGCAGCAGACTTC
	R: TGAGGCGTAATGGCAAACG
NAALADL2	F: CATCGTGCAGTTTGCTTACGA
	R: AACGGGCCTCGGAGAGAA
PLEKHA7	F: GGCGACCTTCCCTAGACCTAA
	R: TCTTGCCTCGCTGGTGATC
SAMNS1	F: GGACAGCTTTCGACTGGATGA
	R: GCACGGCCACAGAATGG
SPRR2A	F: GATGATCCCTGACAGCAAAAAGT
	R: CCACCTGGACAGTGGCAGTA

F, forward; R, reverse.

### Analysis of Kynu Expression

Clone 8 and Scr-control cells were seeded in six-well plates at a density of 5×10^5^cells per well and grown for 24 h. On the following day cells were treated with 1 nM TCDD, 10 µM DIM or dimethyl sulfoxide (DMSO), for 16 hrs. After treatments, cell monolayers were lysed in 1 ml of TRIzol, which allowed for simultaneous isolation of RNA and protein. Total RNA was isolated as described above. Proteins were isolated as previously described [17] and the protein pellets were suspended in RIPA buffer containing 2% SDS and sonicated briefly.

For semi-quantitative RT-PCR, 2 µg of total RNA was reverse transcribed to first strand cDNA, and equivalent of 40 ng was amplified in a 25ul reaction using Bullseye *Taq* DNA polymerase 2X Master Mix (MIDSCI, St. Louis, MO). Ribosomal 18s was amplified for normalization. PCR primers used were: for KYNU (5′ to 3′), CATGCCCATACGATTAAACCTG and CATGCAAGGAACAGACCAACA; for Cyp1a1 (5′ to 3′) TAGACACTGATCTGGCTGCAG and GGGAAGGCTCCATCAGCATC. Immunoblotting was performed as previously described [13]. Primary antibodies used include: anti-AhR and anti- KYNU (Santa Cruz, Dallas, TX), anti-β-Actin (Sigma-Aldrich, St. Louis, MO).

## Results

### Gene Expression Profiling

**Figure 1:**
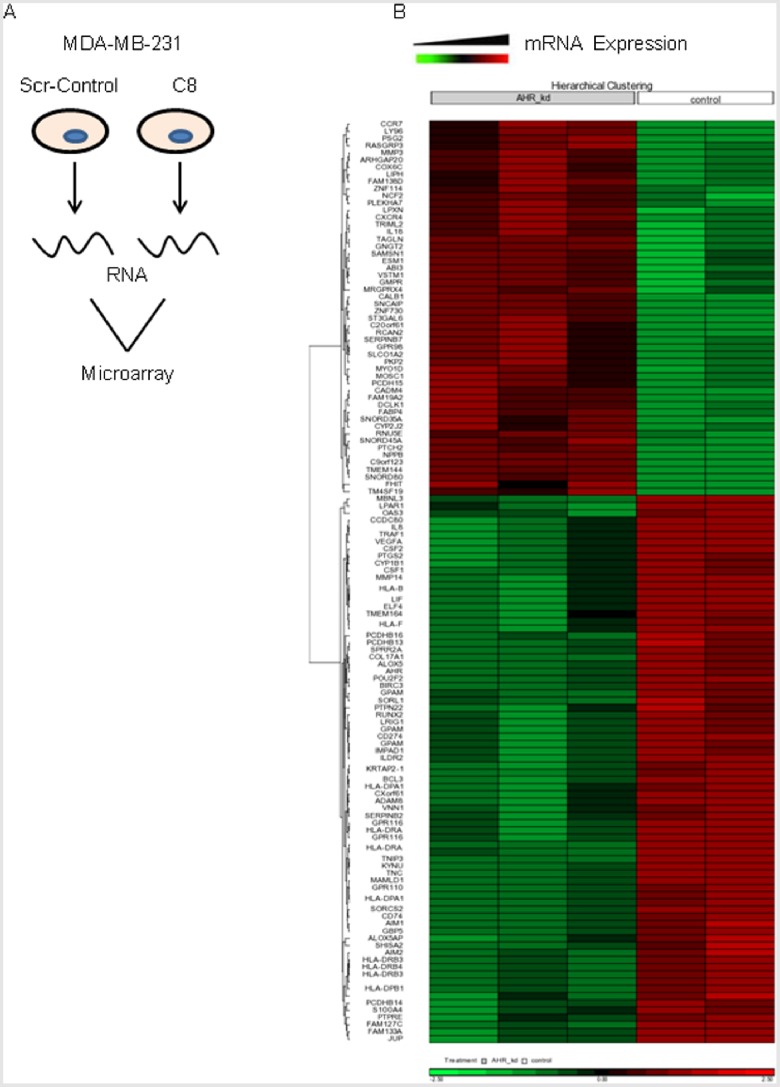
Hierarchical clustering of differentially expressed genes in MDA-MB-231 cells following AhR knockdown. (A) Illustration of the procedure used to examine differential gene expression following AhR knockdown. (B) Clusters of genes altered by AhR KD. Heat map reveals clusters of genes. Green indicates genes up-regulated compared to control cells and red indicates genes down-regulated compared to control cells.

In order to analyze the alterations of transcriptome expression level associated with AhR knockdown, global gene expression profiling was performed on MDA-MB-231 Scr-control cells and clone 8 (C8) of MDA-MB-231 cells with stable AhR knockdown [9]. Profiling analyses identified 308 probe-set level transcripts as being significantly differentially expressed in C8 cells compared with control cells, having a greater than two-fold change in expression and a p-value ≤0.05 (Figure 1). Mapping the probe-sets to known annotated genes yielded 144 significantly changed unique gene identifications. Of the differentially expressed genes, 66 were upregulated >2-fold and 78 were downregulated >2.0-fold in C8 cells. Table 2 summarizes the top ten upregulated or downregulated genes, ordered by the average fold differences (a complete list can be found in Table S1).

**Table 2:** Top 10 genes significantly up- or down-regulated in MDA-MB-231 cells following AhR KD.

Symbol	Description	Fold Change	P-value
**Up**			
CALB1	Calbindin 1, 28kDa	23.4907	2.48E-03
SAMSN1	SAM domain, SH3 domain and nuclear localization signals 1	9.1653	1.70E-02
PLEKHA7	Pleckstrin homology domain containing, family A member 7	8.78089	6.15E-03
MRGPRX4	MAS-related GPR, member X4	8.4079	2.38E-02
NAALADL2	N-acetylated alpha-linked acidic dipeptidase-like 2	8.40242	4.37E-02
PSG2	Pregnancy specific beta-1-glycoprotein 2	7.47155	1.24E-02
IL18	Interleukin 18	7.08637	1.17E-02
DPP4	Dipeptidyl-peptidase 4	6.84522	8.42E-03
MAMDC2	MAM domain containing 2	6.23418	2.94E-02
SNACAIP	Synuclein, alpha interacting protein	6.21389	4.91E-03
**DOWN**			
KYNU	Kynureninase	−11.1561	7.86E-05
SPRR2A	Small proline-rich protein 2A	−8.28175	2.81E-03
CSF1	Colony stimulating factor 1	−6.62339	1.64E-02
HLA-DPA1	Major histocompatibility complex, class II, DP alpha 1	−5.90627	1.09E-02
C15ORF48	Chromosome 15 open reading frame 48	−5.20775	2.63E-02
SERPINB2	Serpin peptidase inhibitor, clade B	−5.06934	4.74E-02
TNIP3	TNF alpha interacting protein 3	−5.00674	2.29E-03
HLA-DRA	Major histocompatibility complex, class II, DR alpha	−5.00129	2.81E-03
IL8	Interleukin 8	−4.69281	4.22E-02
CD74	CD74 molecule, major histocompatibility complex, class I	−4.39972	1.30E-02

### Validation of Microarray Data with Quantitative RT-PCR (qRT-PCR)

**Figure 2:**
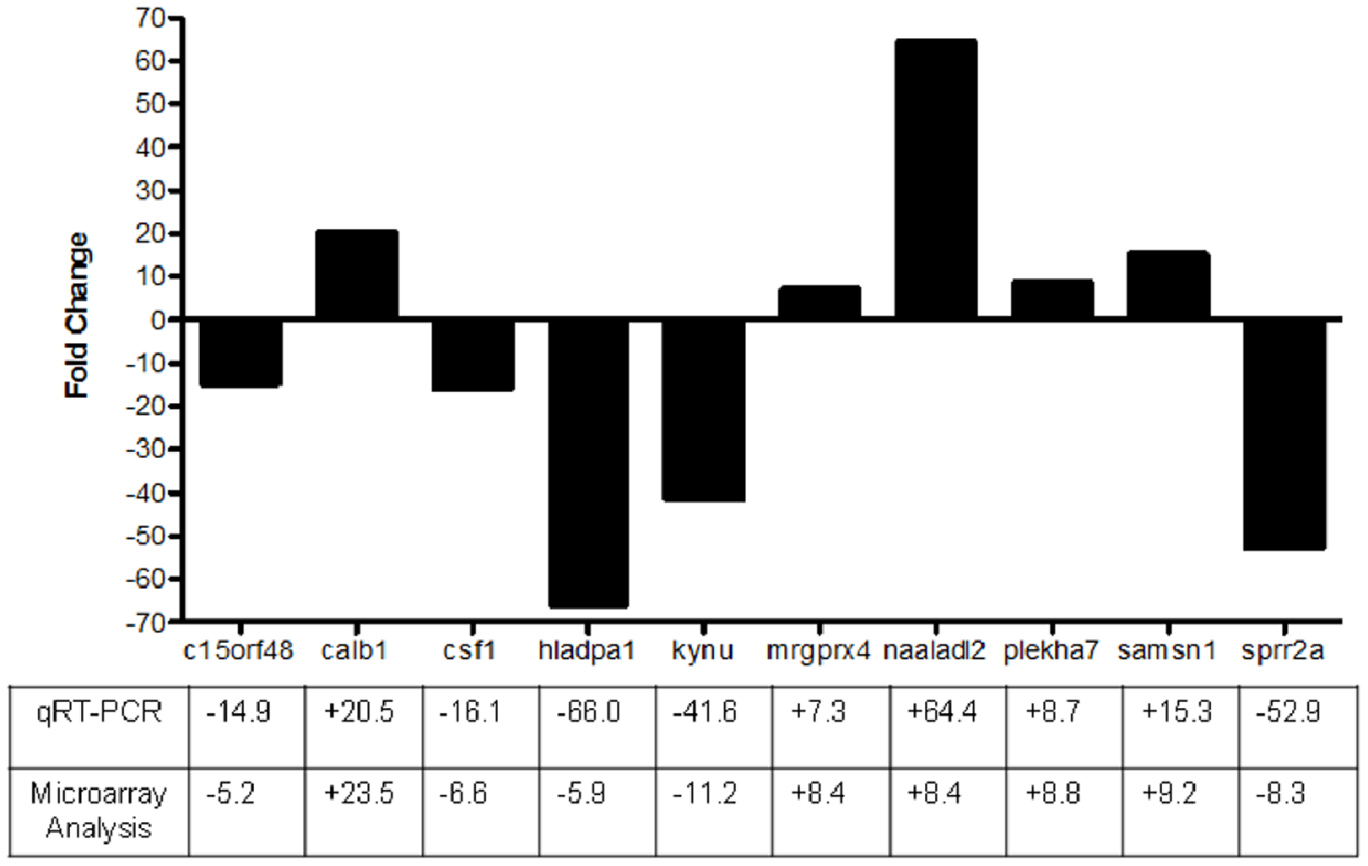
Validation of microarray data by qRT-PCR. Differential gene expression from the microarrays analyses was confirmed by qRT-PCR of 10 selected genes using gene specific primers shown in Table 1. Data represents mean of triplicates, normalized to GAPDH, and presented as relative fold-change (RFC) of Clone 8 to that of Scr-control cells.

Validation of microarray data was done using quantitative RT-PCR (qRT-PCR) on the same RNA samples used for the transcriptome microarray analysis. Ten genes were selected from the 144 differentially expressed genes, choosing the top five upregulated genes (*CALB1, SAMSN1, PLEKHA7, MRGPRX4, NAALADL2*) and the top five downregulated genes (*KYNU, SPRR2A, CSF1, HLA-DPA1, C15orf48*). As shown in Figure 2, the trend (upregulation or down-regulation) in expression of all ten selected genes showed consistency with result from the microarray analysis, validating accuracy of the microarray data.

### Gene Ontology Term and KEGG Pathway Enrichment Analysis

The biological processes involving differential gene expressions were identified by Gene Ontology (GO) enrichment analysis using the Database for Annotation, Visualization and Integrated Discovery (DAVID) tool. In total, 18 GO terms were identified including immune response, response to hypoxia, cell migration, cell adhesion, antigen processing and presentation and angiogenesis (Table 3).

**Table 3:** Functional enrichment of GO biological processes following AhR knockdown in MDA-MB-231 cells (C8).

Accession	Biological Process	Count*	P-value
GO:0006955	Immune response	24	0.0049
GO:0001666	Response to hypoxia	9	0.0116
GO:0016477	Cell migration	14	0.0270
GO:0007155	Cell adhesion	22	0.0270
GO:0019882	Antigen processing and presentation	5	0.0360
GO:0001525	Angiogenesis	9	0.0393

*Counts refer to the number of genes from the input list that fit into the given pathway.

Enrichment was performed using the WebGESTALT online tool. It identified those biological processes that had significant alterations following AhR knockdown.

To identify well characterized molecular pathways that were significantly represented, the genes list was also subjected to pathway analysis using Kyoto Encyclopedia of Genes and Genomes (KEGG). KEGG pathway analysis identified 18 significantly enriched pathways, including those associated with cancer. The following pathways of interest were shown to be significantly enriched: bladder cancer, cytokine-cytokine receptor interaction, mTOR signaling pathway, cell adhesion molecules, toll-like receptor pathway, B cell receptor signaling pathway and VEGF signaling pathway (Table 4). In Table 5, we identified some individual genes that may be associated with the phenotypic changes we previously observed following AhR knockdown [9].

**Table 4:** Functional enrichment of KEGG pathway analysis following AhR knockdown in MDA-MB-231 cells.

Category	Term	Enrichment^1^	P-value^2^
KEGG_Pathway	Allograft rejection	12.03	0.0087
KEGG_Pathway	Antigen processing and presentation	7.38	0.0087
KEGG_Pathway	Graft-versus-host disease	13.00	0.0087
KEGG_Pathway	Type I diabetes mellitus	10.15	0.0087
KEGG_Pathway	Autoimmune thyroid disease	9.28	0.0104
KEGG_Pathway	Bladder Cancer	8.33	0.0126
KEGG_Pathway	Intestinal immune network for IgA production	7.92	0.0133
KEGG_Pathway	Asthma	10.60	0.0188
KEGG_Pathway	Cytokine-cytokine receptor interaction	3.00	0.0188
KEGG_Pathway	mTOR signaling pathway	6.37	0.0188
KEGG_Pathway	Hematopoietic cell lineage	5.01	0.0188
KEGG_Pathway	Cell adhesion molecules (CAMs)	4.03	0.0188
KEGG_Pathway	Axon guidance	3.84	0.0219
KEGG_Pathway	Toll-like receptor signaling pathway	4.41	0.0232
KEGG_Pathway	Viral myocarditis	5.42	0.0232
KEGG_Pathway	NOD-like receptor signaling pathway	5.42	0.0232
KEGG_Pathway	B cell receptor signaling pathway	4.51	0.0387
KEGG_Pathway	VEGF signaling pathway	4.58	0.0387

1 Ratio of enrichment refers to the number of observed genes divided by the number of expected genes from each KEGG category.

2 Multiple test adjustment (BH) FDR adjusted p<0.05 was considered significant.

KEGG pathway analysis was performed using the WebGESTALT online tool. It identified those pathways that had significant alterations following AhR knockdown.

**Table 5:** List of genes related to phenotypic changes observed following AhR knockdown in MDA-MB-231 cells.

Symbol	Description	Cell Process	Fold Change^2^	P-value
KYNU	Kynureninase	Tryptophan Catabolism	−11.15	7.8E-05
MUC1	Mucin 1	Cell Growth	−2.0	0.0412
IL-8	Interleukin 8	Cell Growth	−2.08	1.5E-04
BIRC3	Baculoviral IAP repeat- containing 3	Cell Survival	−3.5	4.1E-04
BCL3	B-cell CLL/lymphoma 3	Cell Survival	−2.98	5.6E-04
ABCC3	ATP-binding cassette, sub-family C. member 3	Multi-drug resistance	−3.29	8.3E-04
S100A4	S100 calcium binding protein A4	Cell Migration and Invasion	−2.86	0.01
ABI3	Abelson interactor protein 3	Cell Migration and Invasion	+5.15	5.7E-04
CCL2	Chemokine (C-C motif) ligand 2	Angiogenesis	−2.7	2.3E-03
VEGFA	Vascular endothelial growth factor A	Angiogenesis	−3.74	9.5E-04

1 P-value <0.05 and fold change ≥2.0 was considered significant.

2 The fold changes in transcriptome levels and significance were calculated using one-way ANOVA.

KEGG pathway analysis was performed using the WebGESTALT online tool. List identified genes which had significant alterations following AhR knockdown^1^.

**Figure 3:**
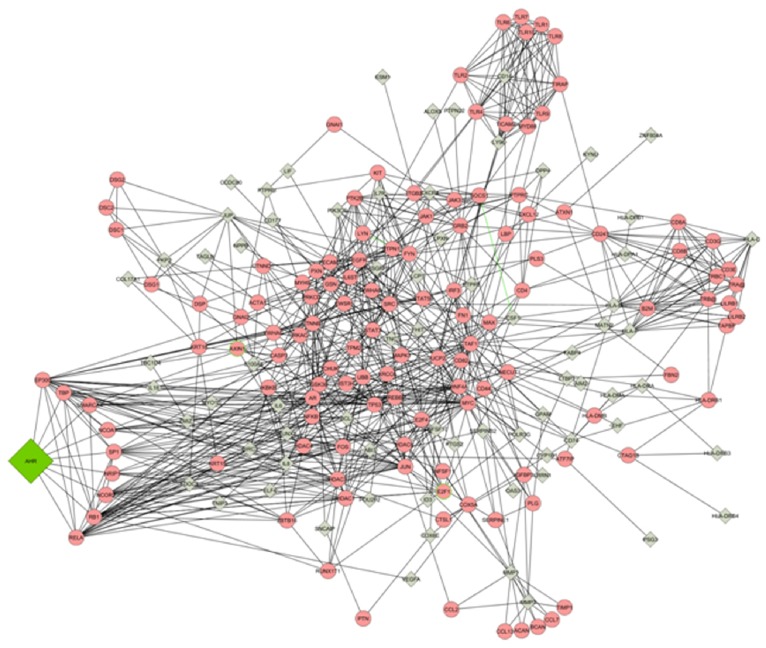
Subnetwork of functional interactions of AhR-target networks. Initial nodes were populated with differential expressed genes. Circles represent genes of AhR targets, while diamonds represent linker genes.

Next, to construct a predicted protein-protein interaction network for interactions between AhR and differentially expressed genes the Michigan Molecular Interactions (MiMI) database was used (Figure 3). The differentially expressed genes were used as the initial population nodes then the network was extended to one degree of biological interaction by known protein interactions from the MiMI database.

### Regulation of KYNU Expression by AhR

**Figure 4:**
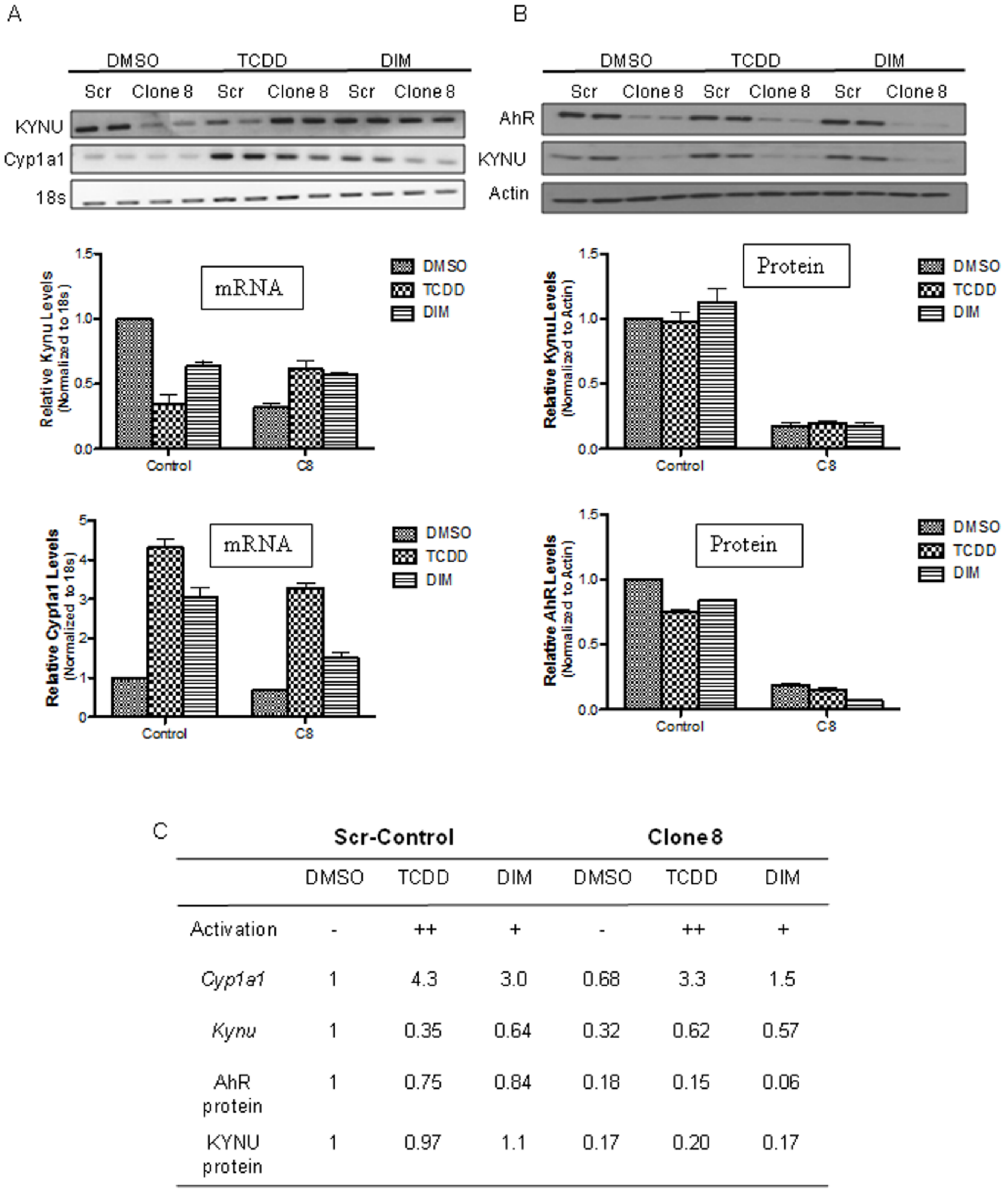
KYNU expression in the Scr-control and Clone 8 cells following treatment with 1 nM TCDD, 10 µM diindolylmethane (DIM) or 0.1% DMSO for 16 h. KYNU expression measured at the mRNA level by RT-PCR (A) and protein level by immunoblot analysis (B) in the presence of vehicle control DMSO or AhR exogenous ligands TCDD and DIM for 16 hrs. Bar graphs are mean ± s.d. from three independent experiments (*p<0.05; **p<0.01). (C) Experimental summary of different treatments relative to DMSO-treated Scr-control cells.

The gene for kynureninase (KYNU), which is an enzyme involved in tryptophan catabolism was notably identified as one of the genes that was considerably downregulated following AhR depletion in C8 cells. Because kynurenine (Kyn), a tryptophan catabolite was recently identified as an endogenous ligand of AhR in neuroblastoma [18], we attempted to define the relevance of the AhR regulation of KYNU, which is the enzyme downstream of the implicated AhR endogenous ligand, Kyn. We examined the effect of activation of AhR by two exogenous ligands, DIM (natural ligand) and TCDD (synthetic ligand) on KYNU expression. Activation of AhR with both TCDD and DIM didn’t affect *KYNU* gene or protein expression significantly in C8 or control cells (Figure 4A & B). Induction of Cyp1a1 expression was measured as a read out of AhR activation. TCDD strongly induced *CYP1A1*, whereas DIM induced *CYP1A1* to a lesser extent in both control and C8 cells (Figure 4A & C). Consistent with the microarray analysis, both *KYNU* gene and protein expression were substantially lower in C8 cells compared to control cells under basal condition (Figure 4 A–C).

## Discussion

Several studies have identified a role for AhR in cancer independent of exogenous ligand. We previously demonstrated that merely reducing AhR expression altered cell proliferation, anchorage independent growth, migration and apoptosis in MDA-MB-231 cells *in vitro*, and reduced orthotopic xenograft tumor growth and experimental pulmonary metastasis *in vivo* [9]. This led us to conclude that AhR has the capacity to influence key cellular process associated with breast cancer aggressiveness (promotion/progression). In order to gain an insight into the mechanisms by which elevated AhR expression influences cancer progression independent of exogenous ligand, this study aimed to identify the alterations of gene expression and possible molecular mechanisms by which AhR overexpression contributes to breast cancer. Utilizing Human Gene 1.0-ST array, we show that AhR knockdown alters the expression of several genes known to be linked with cancer. These genes include those involved in tryptophan metabolism *(KYNU)*, cell growth (*MUC1* and *IL8),* cell survival (*BIRC3* and *BCL3*) cell migration and invasion (*S100A4* and *ABI3),* multi-drug resistance (*ABCC3*) and angiogenesis (*VEGFA* and *CCL2*).

### Tryptophan Catabolism

Tryptophan, an essential amino acid, is metabolized in the local microenvironment of tumors through the kynurenine pathway leading to the production of several catabolites. Recently, kynurenine (kyn), a tryptophan catabolite that is constitutively produced in glioma cells by tryptophan-2,3-dioxygenase (TDO) in amounts sufficient for AhR activation, has been identified as an endogenous ligand of AhR [18]. In our study in the MDA-MB231 breast cancer cell line, the AhR depletion resulted in substantial down-regulation of KYNU, the enzyme which catalyzes the conversion of kyn to anthranilic acid and the conversion of 3-hydroxykynurenine (3HK) to 3-hydroxyanthranilic acid; taking into account that the mammalian KYNU preferentially targets the 3HK over kyn [19]. The lack of the effect of the AhR exogenous ligands on *KYNU* gene expression suggests that its’ expression is modulated by AhR levels rather than AhR ligand-dependent activation. Furthermore, the lack of a putative XRE within the KYNU promoter suggests that AhR modulates *KYNU* gene expression either by binding to a novel non consensus XRE or through interaction with other transcription factors, possibly serving as a co-activator. Down-regulation of KYNU subsequent to AhR depletion could potentially lead to increased accumulation of 3HK, which may induce apoptosis through increased generation of ROS [18], [20]. This could partially account for the increased apoptosis previously observed *in vitro* and *in vivo* following AhR knockdown [9].

### Cell Growth and Survival

Aggressive cell growth is a well defined characteristic of cancer cells. *MUC1* and *IL8* expressions were downregulated by AhR knockdown in MDA-MB 231. These genes are associated with cell growth, and therefore, their downregulated expression may contribute to the previously observed reduced proliferation of these cells following AhR knockdown [9]. Mucin 1 (MUC1) is a tumor associated glycoprotein that plays a role in cancer progression. MUC1 promotes the transcription of *MAP2K1* (MEK1), *JUN*, *PDGFA*, *CDC25A*, *VEGF* and *ITGAV* (integrin α_v_); genes involved in proliferation and cell survival, by affecting the recruitment of co-activators [21]. Interleukin-8 (IL-8) is a CXC-type chemokine that has multiple functions within the tumor microenvironment; promoting cell growth, invasion and metastasis of cancer cells through binding its receptors, CXCR1 and CXCR2 in an autocrine fashion [22], [23]. In many cancers MUC1 and IL-8 are overexpressed and are associated with poor prognosis [21], [22].

Cancer cells often gain resistance to apoptosis providing the basis for cell survival and growth by the expressing anti-apoptotic proteins. AhR knockdown resulted in down-regulation of the expression of two anti-apoptotic genes, *Baculoviral IAP repeat containing 3 (BIRC3) and B-cell CLL/lymphoma 3 (BCL3).* Both *BIRC3* and *BCL3* function to inhibit apoptosis and their elevated expression has been observed in a number of cancers [24]–[26]. Therefore, the downregulation of these genes may also contribute to the increased apoptosis previously observed in C8 cells compared to control cells.

### Multi-Drug Resistance

The development of multi-drug resistance (MDR) remains a major obstacle in the chemotherapy of breast cancer and can develop by increased drug efflux via ATP-binding cassette (ABC). AhR knockdown downregulated the expression of *ABCC3*, which is a member of the *ABC* gene family ABCC3 overexpression is observed in breast cancer and has been implicated in acquired MDR [27]. Considering ABCC3 has been identified as conferring resistance to paclitaxel [28], a chemotherapeutic agent frequently used in the treatment of metastatic breast cancer, the downregulation of its expression may contribute to the sensitization of MDA-MB-231 cells to paclitaxel we previously observed following AhR knockdown [9].

### Cell Migration and Invasion

Our analysis also revealed a down-regulation in the expression of *S100 calcium binding protein A4 (S100A4)* and upregulation of *abelson interactor protein 3 (ABI3)* genes. S100A4 is associated with cell migration and invasion; key steps in cancer metastasis. It functions by interacting with target proteins involved in cytoskeleton rearrangement and cell motility in a calcium-dependent manner, including F-actin, tropomyosin, and the heavy chain of non-muscle myosin II [29]. Notably, S100A4 is overexpressed in metastatic cancers and is characterized as a marker of tumor progression [30]. On the other hand, ABI3 is a component of the Abi/WAVE complex and it regulates Rac-dependent actin polymerization and formation of lamellipodia. ABI3 functions as a tumor suppressor inhibiting ectopic metastasis of tumor cells and its expression is often lost in invasive cancer cell lines [31], [32]. Therefore altered expression of both these genes may contribute to the decreased metastatic potential of MDA-MB-231 cells following AhR knockdown [9].

### Angiogenesis

As angiogenesis has a critical role in the metastatic process, which is the primary cause of mortality in cancer patients, the down-regulation of *chemokine (C-C motif) ligand 2 (CCL2) and vascular endothelial growth factor A (VEGFA),* subsequent to AhR knockdown was deemed of high significance. CCL2 is a chemokine that functions as a chemoattractant, facilitating angiogenesis through the recruitment of tumor associated macrophages (TAMs). TAMs promote a microenvironment that supports tumor growth and metastasis through the production of growth and angiogenic factors, including VEGFA [33], [34]. CCL2 can also recruit endothelia cells, which express a CCL2 receptor [35]. VEGFA, a cytokine that is a major regulator of angiogenesis in cancer is secreted by cancer cells under hypoxic conditions and leads to recruitment of endothelial cells through binding to its receptor [36]. Therefore downregulated expression of *CCL2* and *VEGFA* following AhR knockdown may decrease the blood supply necessary for tumor growth. In our previous studies [9] we have not examined closely the impact of AhR depletion on the angiogenesis process, it will be of interest to perform angiogenesis assays *in vivo* to verify the relationship between AhR level and development of angiogenesis.

## Conclusion

**Figure 5:**
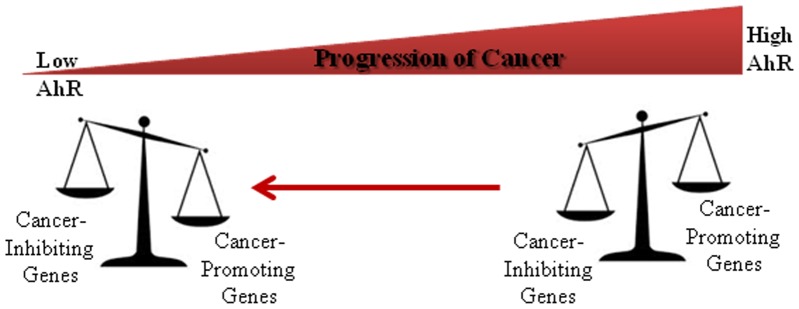
A schematic model depicting how reduction of AhR can alter the expression of genes responsible for promoting or inhibiting cancer progression. Reduced AhR expression directly or indirectly alters the expression of genes regulating tumor growth and survival, favoring a state that opposes tumor progression.

As many of these genes are multifunctional and the primary cause for cellular transformation is aberrant expression of genes/proteins, this study sheds light on molecular mechanisms through which AhR overexpression may influence breast cancer progression. The fact that AhR knockdown alters various genes involved in different biological processes suggests that the role of AhR in breast cancer merits further investigation. The protein–protein interaction network (PIN) (Figure 3) provides insight on how AhR expression might influence breast cancer. The PIN suggests that AhR transcriptional activity may be modified through direct interaction with transcription factors (TBP and SP1), co-activators (EP300 and NCOA1) and co-repressors (NRIP1 and NCOR2). In addition, the network links AhR with proteins involved in cellular growth, cellular migration, immune response and gene regulation. Analysis examining protein levels will be crucial, as studies have shown that mRNA levels often do not correlate with protein levels. Nevertheless, the identification of these genes has provided new insight into possible molecular events affected by reducing AhR expression that could explain the phenotypic changes we previously observed *in vitro* and *in vivo* [9]. In these studies, we showed that the depletion of AhR in metastatic MDA-MB-231 remarkably attenuated their tumorigenic growth *in vitro* and *in vivo*, as well as inhibited their lung metastasis in nude mouse model. Taken together with the sets of genes we identified in this study, we can conclude that AhR knockdown alters the expression of genes enhancing or inhibiting cancer progression; tipping the balance towards a state that counteracts tumor progression (Figure 5).

## Supporting Information

Table S1 Complete List of Transcriptome Microarray Analysis of AhR KD clone 8 MDA-MB-231 cells compared to scramble control MDA-MB-231 cells.
(XLSX)
